# How Interactions during Viral–Viral Coinfection Can Shape Infection Kinetics

**DOI:** 10.3390/v15061303

**Published:** 2023-05-31

**Authors:** Lubna Pinky, Joseph R. DeAguero, Christopher H. Remien, Amber M. Smith

**Affiliations:** 1 Department of Pediatrics, University of Tennessee Health Science Center, Memphis, TN 38163, USA; 2 Bioinformatics and Computational Biology Program, University of Idaho, Moscow, ID 83844, USA; 3 Department of Mathematics and Statistical Science, University of Idaho, Moscow, ID 83844, USA

**Keywords:** viral coinfection, influenza, RSV, rhinovirus, SARS-CoV-2, viral dynamics, mathematical modeling

## Abstract

Respiratory viral infections are a leading global cause of disease with multiple viruses detected in 20–30% of cases, and several viruses simultaneously circulating. Some infections with unique viral copathogens result in reduced pathogenicity, while other viral pairings can worsen disease. The mechanisms driving these dichotomous outcomes are likely variable and have only begun to be examined in the laboratory and clinic. To better understand viral–viral coinfections and predict potential mechanisms that result in distinct disease outcomes, we first systematically fit mathematical models to viral load data from ferrets infected with respiratory syncytial virus (RSV), followed by influenza A virus (IAV) after 3 days. The results suggest that IAV reduced the rate of RSV production, while RSV reduced the rate of IAV infected cell clearance. We then explored the realm of possible dynamics for scenarios that had not been examined experimentally, including a different infection order, coinfection timing, interaction mechanisms, and viral pairings. IAV coinfection with rhinovirus (RV) or SARS-CoV-2 (CoV2) was examined by using human viral load data from single infections together with murine weight-loss data from IAV-RV, RV-IAV, and IAV-CoV2 coinfections to guide the interpretation of the model results. Similar to the results with RSV-IAV coinfection, this analysis shows that the increased disease severity observed during murine IAV-RV or IAV-CoV2 coinfection was likely due to the slower clearance of IAV-infected cells by the other viruses. The improved outcome when IAV followed RV, on the other hand, could be replicated when the rate of RV infected cell clearance was reduced by IAV. Simulating viral–viral coinfections in this way provides new insights about how viral–viral interactions can regulate disease severity during coinfection and yields testable hypotheses ripe for experimental evaluation.

## 1. Introduction

During a respiratory infection, multiple viruses may be present and working in concert to cause disease [1,2,3,4,5]. Several respiratory viruses, including rhinovirus (RV), respiratory syncytial virus (RSV), influenza A and B viruses (IAV and IBV), human metapneumovirus (HMPV), human parainfluenza viruses (PIV), adenoviruses (ADV), and coronaviruses (CoVs), have been found concurrently within hosts with pneumonia [6,7,8,9,10,11,12,13,14,15,16,17,18,19,20]. Children are more likely to be concurrently infected with multiple respiratory viruses, where up to 30% have more than one respiratory virus present when admitted to the hospital for severe clinical disease [16,19,21,22,23,24,25]. Patients with coinfection have shown diverse disease outcomes that ranged from mild to severe, with severity increasing compared to that of patients who were infected with a single virus.

Data on viral–viral coinfections are somewhat limited, but some studies have begun evaluating the outcomes of coinfection with commonly observed viral pairs (e.g., IAV and RSV [26,27,28,29,30,31], IAV and PIV [32], IAV and RV [28,33,34,35], RSV and RV [28], RSV and HMPV [36], IAV and severe acute respiratory syndrome (SARS)-CoV-2 [37,38,39,40,41,42,43], RSV and SARS-CoV-2 [43], and RV and SARS-CoV-2 [44]). Collectively, these studies observed diverse outcomes of viral–viral coinfections with some interactions resulting in enhanced spread of one or both viruses within the respiratory tract, while other mechanisms seem to work in an inhibitory manner. For example, PIV-2 enhanced cell-to-cell fusion through the expression of its surface glycoproteins, which boosted viral spread between cells, and increased IAV titers but not PIV titers [32]. On the other hand, infection with IAV limited a concurrent RSV infection by promoting intracellular competition for proteins or amino acids needed for the successful replication of both viruses within cell cultures [11,26]. In vivo infections in animal models support the exclusion of RSV by IAV and suggest that RSV prior to IAV decreases disease severity [27,29,30]. A similar competitive exclusion was observed in 3D tissue cultures during coinfection with RSV and HMPV, where HMPV was inhibited without any effect on RSV [36]. The reduction was accompanied by higher Type I and III interferon (IFN) responses [36]. IFN-mediated effects were also implicated in RV–IAV coinfection where RV-induced IFN protected against subsequent IAV infection within differentiated airway cell cultures [34].

The relative timing between viruses and the order in which the viruses infect the host seem to contribute to differing disease outcomes [27,29,33,37,39,40,42]. Interestingly, the exclusion effect during RSV–HMPV coinfection was more robust during a concurrent infection compared to that in an infection where HMPV followed RSV after 2 days. This is in contrast to other viral–viral coinfections where infections separated by 2 to 5 days had more robust effects [33,37,42]. For example, RV attenuated IAV-mediated disease severity and reduced IAV titers when RV infection had occurred 2 days before IAV, but the effect was reduced during simultaneous infection [33]. Conversely, animals coinfected with IAV 2 days before RV experienced greater disease severity [33]. Similar outcomes occurred in animals coinfected with IAV 3 days before SARS-CoV-2 [37].

These empirical studies illuminate the breadth of interactions that lead to diverse outcomes of respiratory viral–viral coinfections and the need for mathematical methods that could dissect complex, time-dependent, and potentially nonlinear mechanisms, as they do for viral–bacterial coinfection [45]. One study on viral–viral coinfections suggested that a faster-replicating virus would outcompete other viruses in a scenario where two viruses were competing for epithelial cells [46]. However, it is possible that viruses infect different cells or infect different areas of the respiratory tract. In addition, as noted above, they may inhibit or enhance other processes (e.g., replication rates and/or immune responses) and ultimately modulate disease. Thus, expanded modeling infrastructures are needed and are a focus of this study. Unfortunately, most studies on viral–viral coinfections lack quantitative information on viral loads and/or host immune responses that are needed to effectively use mathematical approaches, which typically include fitting a mechanistic model to data. However, weight loss, which is a measure of disease severity, is tracked in most murine studies, and our recent work showed that mathematical models can accurately connect animal weight loss to infection kinetics during monoinfections and viral–bacterial coinfections [47,48]. These links allow for us to better interpret weight-loss data, and afford the ability to assess mechanisms with limited data to modeling studies such as this one.

To address gaps in understanding viral–viral coinfections, we assessed RSV, RV, and SARS-CoV-2 coinfections with IAV using two mathematical models. We predicted potential underlying mechanisms of viral interference and cooperation and assessed how different viral orders, timings, and pairings affected the infection dynamics and disease severity. The results provide important insights into divergent outcomes, in addition to generating novel hypotheses regarding why certain viral orders enhance or reduce disease severity.

## 2. Materials and Methods

### 2.1. Data for RSV-IAV Coinfection in Ferrets

Viral-load data were digitized from a study where ferrets were monoinfected or coinfected with a long strain of RSV and/or influenza A/Tasmania/2004/2009 (A[H1N1]pdm09; IAV) [27]. Briefly, groups of 4 ferrets were intranasally infected with $3.5\;\log_{10}$ 50% tissue culture infectious dose (${\mathrm{TCID}}_{50}$) of IAV, $5.0\;\log_{10}$ plaque-forming units (PFU) of RSV, or IAV followed 3 days later by RSV. Viral RNA copy number per 100 μL of nasal wash was measured daily for 14 days post infection (pi).

### 2.2. Data for IAV, RV, and SARS-CoV-2 Infections in Humans

Viral-load data were digitized from studies where humans were experimentally or naturally monoinfected with IAV [49], RV [50], or SARS-CoV-2 [51]. For each, we chose one patient for ease of investigating potential coinfection dynamics. For IAV, human volunteers were experimentally infected intranasally with $4.2\;\log_{10}\;{\mathrm{TCID}}_{50}$ of influenza A/Hong Kong/123/77. Nasal washes were collected daily for 7 days, and infectious viral titers were determined via ${\mathrm{TCID}}_{50}$. The used data here were from Patient 4 due to this individual having clear viral growth, peak, and decay phases. For RV [50], 14 human volunteers were intranasally infected with $2.4\;\log_{10}\;{\mathrm{TCID}}_{50}$ of RV, which was reported as the geometric mean. Nasal washes were collected daily for 5 days. For SARS-CoV-2 [51], the data were from naturally infected patients. Viral loads were sampled from throat swabs and measured in RNA copies/mL. All samples were taken approximately 2–4 days after the symptoms. We used Patient 8 due to their clear viral-load dynamics, and assumed that the infection had initiated 5 days before the onset of symptoms.

### 2.3. Data for IAV Coinfection with RV or SARS-CoV-2 in Mice

Weight-loss data were digitized from a study where BALB/c mice had been intranasally infected with $7.6\times 10^{6}\;{\mathrm{TCID}}_{50}$ of RV1B and/or $100\;{\mathrm{TCID}}_{50}$ of influenza A/Puerto Rico/8/1934 (PR8) [33]. Coinfections were initiated simultaneously or sequentially at a 2 day intervals (IAV-RV or RV–IAV) [33]. Weight loss was measured daily for 14 days.

Weight-loss data were digitized from a study where K18-hACE2 mice had been intranasally infected with $1\times 10^{2}\;\mathrm{PFU}$ of influenza A/HKx31 (H3N2) and/or $1\times 10^{4}\;\mathrm{PFU}$ of hCoV-2/human/Liverpool/REMRQ0001/2020 (SARS-CoV-2) [37]. Coinfections were examined where IAV was given first followed by SARS-CoV-2 after 3 days. Weight loss was measured daily for 10 days.

### 2.4. Mathematical Model of Viral Monoinfection

To describe the dynamic interactions between epithelial cells and the virus during monoinfection, we used the viral kinetic model in Equations (1)–(4) (reviewed in [45,52]). Briefly, in the model, target (epithelial) cells (*T*) are infected by the virus (*V*) at a rate βV per day. Once virus is internalized, the cell undergoes an eclipse phase (*E*) during which infected cells do not yet produce the virus. The cells then transition to the infectious phase (*I*) at a *k* rate per day. Productively infected cells are cleared at a δ rate per day. The virus is produced at a *p* rate per cell per day and cleared at a *c* rate per day.

(1) $$\begin{array}{cc} \frac{dT}{dt} & =-\beta TV \end{array}$$

(2) $$\begin{array}{cc} \frac{dE}{dt} & =\beta TV-kE \end{array}$$

(3) $$\begin{array}{cc} \frac{dI}{dt} & =kE-\delta I \end{array}$$

(4) $$\begin{array}{cc} \frac{dV}{dt} & =pI-cV \end{array}$$

### 2.5. Mathematical Model of Viral–Viral Coinfection

#### 2.5.1. Target Cell Competition

**Figure 1:**
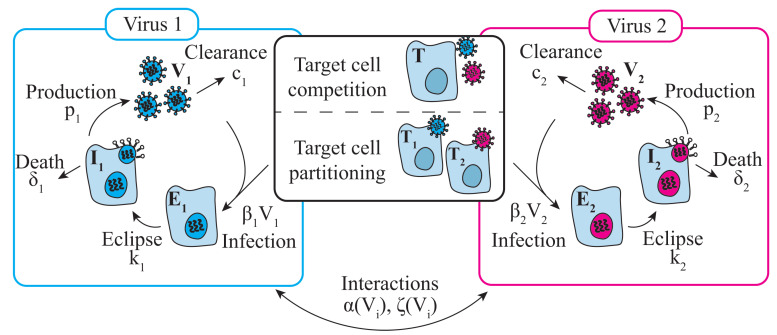
**Coinfection model schematic.** Schematic of the coinfection models in Equations (5)–(8) and Equations (9)–(12). In the ‘target cell competition’ model, two viruses ($V_{1,2}$) interact indirectly by competing for target cells (*T*) [46]. In the ‘target cell partitioning’ model, two viruses do not interact, and each has their own pool of target cells ($T_{1,2}$). In each model, target cells become infected by the virus at rates $\beta_{i}V_{i}$, where subscript i=1,2 denotes the specific rates to $V_{1}$ and $V_{2}$, respectively. Infected cells enter an eclipse phase ($E_{i}$) and transition to producing the virus at rate $k_{i}$. Productively infected cells ($I_{i}$) produce the virus at rate $p_{i}$ and are cleared at rate $\delta_{i}$. The virus is cleared at rate $c_{i}$. Direct interactions were implemented by increasing and/or decreasing one or more of the rates using functions $\alpha (V_{i})$ and/or $\zeta (V_{i}$), respectively, due to the other virus.

To model viral–viral coinfections, we expanded the model in Equations (1)–(4) using two hypotheses (Figure 1). The first hypothesis assumed that two viruses ($V_{1}$ and $V_{2}$) competed for target cells (*T*) (‘target cell competition model’; Equations (5)–(8)) [46]. In this model, a single equation for target cells (*T*) was used where each virus could infect these cells at rates $\beta_{1}V_{1}$ per day and $\beta_{2}V_{2}$ per day. All other equations were equivalent to those in the monoinfection model. Subscripts i=1,2 denote each virus.

(5) $$\begin{array}{cc} \frac{dT}{dt} & =-\beta_{1}TV_{1}-\beta_{2}TV_{2} \end{array}$$

(6) $$\begin{array}{cc} \frac{dE_{i}}{dt} & =\beta_{i}TV_{i}-k_{i}E_{i} \end{array}$$

(7) $$\begin{array}{cc} \frac{dI_{i}}{dt} & =k_{i}E_{i}-\delta_{i}I_{i} \end{array}$$

(8) $$\begin{array}{cc} \frac{dV_{i}}{dt} & =p_{i}I_{i}-c_{i}V_{i} \end{array}$$

#### 2.5.2. Target Cell Partitioning

The second hypothesis assumed that each virus had its own pool of epithelial cells to infect ($T_{1}$ and $T_{2}$) because viruses may preferentially infect certain cell types [53,54,55,56,57] or be present in a different areas of the respiratory tract (‘target cell partitioning model’; Equations (9)–(12)). All other equations remained the same but were distinct for each virus, resulting a total of 8 equations. Subscripts i=1,2 denote each virus.

(9) $$\begin{array}{cc} \frac{dT_{i}}{dt} & =-\beta_{i}T_{i}V_{i} \end{array}$$

(10) $$\begin{array}{cc} \frac{dE_{i}}{dt} & =\beta_{i}T_{i}V_{i}-k_{i}E_{i} \end{array}$$

(11) $$\begin{array}{cc} \frac{dI_{i}}{dt} & =k_{i}E_{i}-\delta_{i}I_{i} \end{array}$$

(12) $$\begin{array}{cc} \frac{dV_{i}}{dt} & =p_{i}I_{i}-c_{i}V_{i} \end{array}$$

#### 2.5.3. Modeling Viral–Viral Interactions

To assess the effect of one virus on another, we used functions that enhanced ($\alpha (V_{i})$ (Equation (13)) or inhibited ($\zeta (V_{i})$; Equation (14)) a particular infection process (i.e., rates of viral infection ($\beta_{i}$), viral production ($p_{i}$), infected cell clearance ($\delta_{i}$), or viral clearance ($c_{i}$)).

(13) $$\begin{array}{ccc} \alpha (V_{i}) & =1+\kappa V_{i} & \end{array}$$

(14) $$\begin{array}{cc} \zeta (V_{i}) & =\frac{1}{1+\kappa V_{i}} \end{array}$$

Parameter κ (per RNA/mL or TCID${}_{50}$/mL) is the strength of the interaction.

### 2.6. Quantifying the Relative Change in Total Virus

To quantify changes in the viral loads and the total viral burden as a consequence of an interaction during coinfection, we calculated the relative change in total virus (i.e., the area under the curve (AUC)) of viral load using Equation (15),

(15) $$\begin{array}{c} \Delta V_{\mathrm{AUC}}\;=\frac{V_{\mathrm{AUC}}^{\mathrm{coinf}}-V_{\mathrm{AUC}}^{\mathrm{single}}}{V_{\mathrm{AUC}}^{\mathrm{single}}}, \end{array}$$

where $V_{\mathrm{AUC}}^{\mathrm{coinf}}$ is the AUC for the coinfection and $V_{\mathrm{AUC}}^{\mathrm{single}}$ is the AUC for the monoinfection. The AUC was calculated using the Python function *scipy.integrate.trapz*.

### 2.7. Quantifying Disease Severity

To quantify the percentage of the lung infected by the virus that related to animal weight loss [47], we calculated the cumulative area under the curve (CAUC) of the infected cell dynamics [47] using the Python function *scipy.integrate.cumtrapz*.

### 2.8. Parameter Estimation

For model fits to the ferret data, parameters were estimated using a nonlinear mixed-effect modeling (NLME) and stochastic approximation expectation minimization (SAEM) algorithm implemented in Monolix 2019R1 [58]. In the NLME approach, each individual parameter is drawn from a log-normal distribution and written as $\theta_{i}=\theta e^{\eta_{i}}$, $\eta_{i}=N\left(0,\omega_{i}^{2}\right)$, where θ denotes the median value of the parameter in the population, and $\eta_{i}$ denotes the random effect that accounts for the interindividual variability of the parameter within the population. Interindividual variability was allowed for all the estimated parameters with the assumption of no correlation and applying an additive residual error model for $\log_{10}$ viral loads. For model fits to the human data, parameters were estimated using scipy.optimize.minimize in Python. Here, only a single patient monoinfected with a virus was used.

The initial number of target cells ($T_{0}$) was set to $5\times 10^{7}\;\mathrm{cells}$ for ferrets and $2\times 10^{8}\;\mathrm{cells}$ for humans. Similar to our previous studies [47,59,60], we fixed the initial number of infected cells ($E_{0}$) to $3.1\times 10^{3}$ cells for IAV infection and $1.0\times 10^{5}$ cells for RSV infection in ferrets [27] and $1\times 10^{2}$ cells for all infections in humans. We considered other values of $E_{0}$ and found no significant differences in estimated parameters, which is consistent with our prior studies [47,59,60]. The initial number of productively infected cells ($I_{0}$) and the initial free virus ($V_{0}$) were set to 0.

The duration of the eclipse phase (1/k) for each virus was kept within a biologically feasible value, and set to 4.8 h for IAV and to 8.0 h for RSV, RV, and SARS-CoV-2. For the monoinfection model (Equations (1)–(4)), estimated parameters included the rates of viral infection (β), viral production (*p*), viral clearance (*c*), and infected cell clearance (δ). The rate of viral infection (β) was allowed to vary between $1\times 10^{-9}$ and 1.0 RNA${}^{-1}$ d${}^{-1}$ or TCID${}_{50}^{-1}$ d${}^{-1}$, and the rate of viral production (*p*) was allowed to vary between $1\times 10^{-3}$ and $1\times 10^{3}$ RNA/cell/d or TCID${}_{50}$/cell/d. The rate of infected cell clearance (δ) was given a lower limit of $1\times 10^{-2}$ d${}^{-1}$ and an upper limit of $1\times 10^{3}\;d^{-1}$. For the coinfection models, we first simulated the monoinfection until the day of coinfection and then employed the coinfection models (Equations (5)–(8) or Equations (9)–(12)) while incorporating the enhancement or inhibition functions (Equations (13) and/or (14)). The strength of interaction (κ) was estimated for each scenario.

Fit quality was assessed using the Akaike information criterion with small sample size correction (${\mathrm{AIC}}_{c}$). The model with the lowest ${\mathrm{AIC}}_{c}$ was considered the best, and $\Delta {\mathrm{AIC}}_{c}\leq 2$ was considered statistically equivalent [61].

## 3. Results

### 3.1. Model-Predicted Mechanisms of RSV–IAV Coinfection

**Figure 2:**
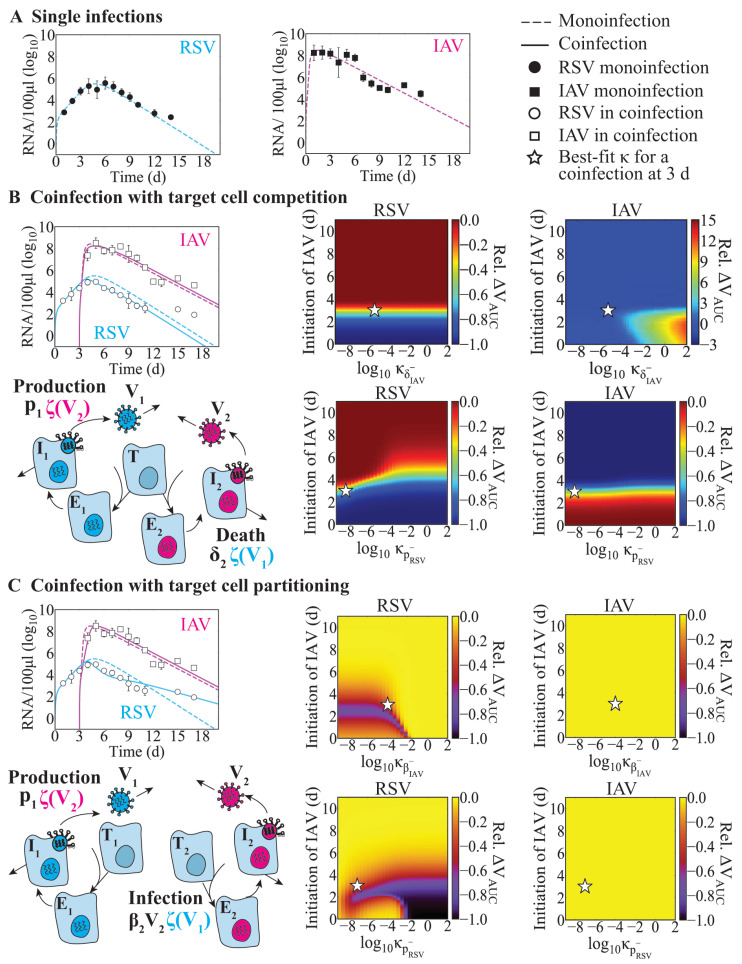
**Fit of the RSV–IAV coinfection models.** (**A**) Fit of the single virus model (Equations (1)–(4)) to viral titers from ferrets infected with IAV (black squares) or RSV (black circles). (**B**,**C**) Comparison of the single infection model fit (dashed lines) and fit of the coinfection models (solid lines; (**B**) Equations (5)–(8) or (**C**) Equations (9)–(12) with the interaction functions (Equations (13) and (14)) to viral titers from ferrets infected with RSV followed by IAV after 3 days (IAV, white squares; RSV, white circles). Dynamics of the (**B**) target cell competition model ($\delta_{\mathrm{IAV}}^{-}$ and $p_{\mathrm{RSV}}^{-}$) or (**C**) target cell partitioning model ($\beta_{\mathrm{IAV}}^{-}$ and $p_{\mathrm{RSV}}^{-}$) are shown along with the model schematic. Heatmaps are the relative change in total viral burden (i.e., $\Delta V_{\mathrm{AUC}}$; Equation (15)) evaluated for a range of interaction strengths (κ = $1\times 10^{-9}$ to $1\times 10^{2}$ (RNA/100 μL)${}^{-1}$) and infection intervals (0 to 11 days). The best-fit κ for a coinfection at 3 days is denoted by a white star.

We used data from ferrets infected with a long strain of RSV and/or influenza A/Tasmania/2004/2009 (A[H1N1]pdm09; IAV) that had their viral loads measured until 14 days post infection (pi) [27]. When ferrets were inoculated with RSV followed by IAV 3 days later, morbidity was reduced, and IAV titers were slightly lower. To begin examining the interactions between RSV and IAV during coinfection that resulted in these dynamics, and establish the baseline parameter values for use in our mathematical models, we first fit the monoinfection model (Equations (1)–(4)) to the data from IAV- or RSV-infected ferrets (Table 1, Figure 2A). This showed a robust fit to each data set and yielded a faster rate of infection ($\beta_{\mathrm{RSV}}=1.0\;\times \;10^{-5}$ (RNA/100 μL)${}^{-1}$ d${}^{-1}$ vs. $\beta_{\mathrm{IAV}}=3.3\;\times \;10^{-6}$ (RNA/100 μL)${}^{-1}$ d${}^{-1}$ [IAV]) and slower rate of viral production for RSV ($p_{\mathrm{RSV}}=3.0\;\times \;10^{-2}$ RNA/100 μL/cell/d vs. $p_{\mathrm{IAV}}=2.5\;\times \;10^{1}$ RNA/100 μL/cell/d).

Using the single infection parameters, we simulated the ‘target cell competition’ model (Equations (5)–(8)) and the ‘target cell partitioning’ model (Equations (9)–(12)) (Figure 1) by first assuming that there were no direct interactions (κ=0). Under this assumption, the target cell partitioning hypothesis performed better than the target cell competition hypothesis (i.e., lower AIC${}_{c}$; 185.3 (‘partitioning’) vs. 189.0 (‘competition’); Table 2), but the data were not precisely replicated.

**Figure A1:**
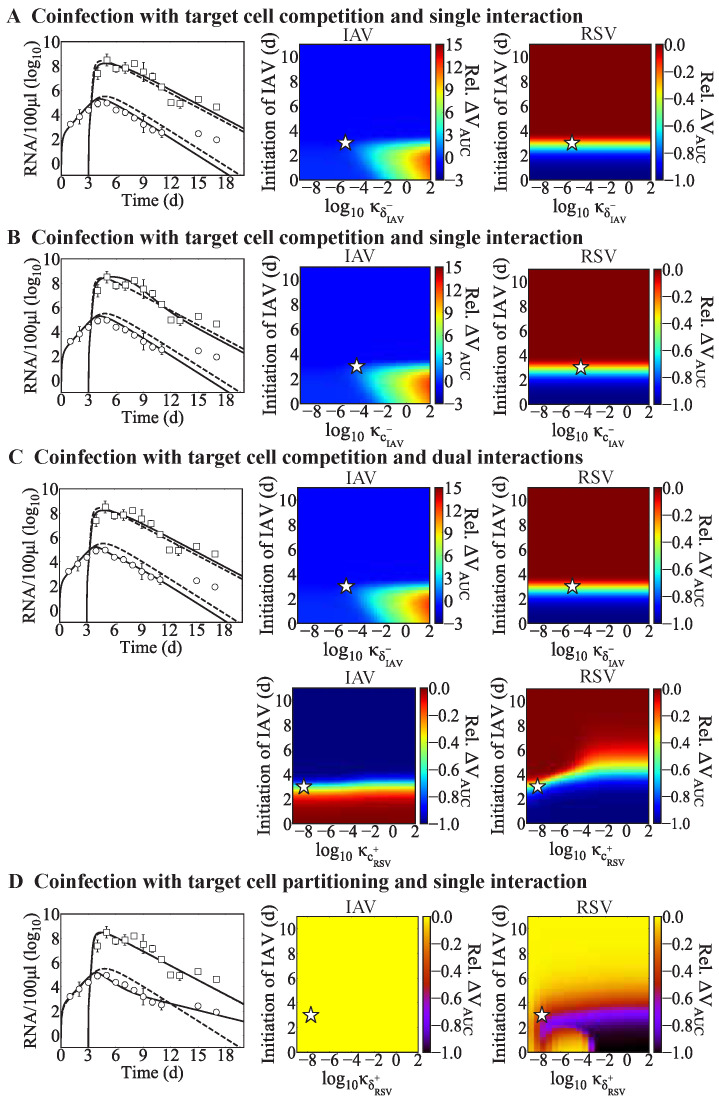
**Model dynamics of alternate mechanisms of RSV-IAV coinfection.** Comparison of the monoinfection model fit (dashed lines; Equations (1)–(4)) and fit of the coinfection models (solid lines; Equations (5)–(8) or Equations (9)–(12)) with the interaction functions (Equations (13) and (14)) to viral titers from ferrets infected with RSV followed by IAV after 3 days (RSV, white circles; IAV, white squares). Heatmaps are the relative change in total viral burden (i.e., $\Delta V_{\mathrm{AUC}}$; Equation (15)) evaluated for a range of interaction strengths (κ = $1\times 10^{-9}$ to $1\times 10^{2}$ RNA/100 μL) and infection intervals (0 to 11 d). The best-fit κ for a coinfection at 3 days is denoted by a white star. Model dynamics of the target cell competition model with (**A**) $\delta_{\mathrm{IAV}}^{-}$, (**B**) $c_{\mathrm{IAV}}^{-}$ (bottom), (**C**) $\delta_{\mathrm{IAV}}^{-}$ and $c_{\mathrm{RSV}}^{+}$. (**D**) Model dynamics of the target cell partitioning model with $\delta_{RSV}^{+}$.

**Figure A2:**
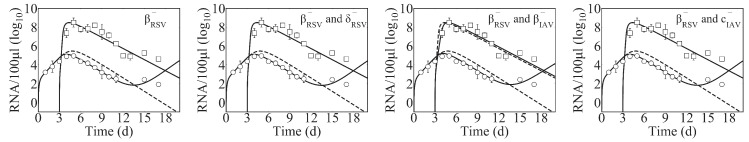
**Model dynamics excluded due to a predicted rebound of RSV during RSV-IAV coinfection.** Fit of the monoinfection model (dashed lines; Equations (1)–(4)) or the target cell partitioning model with the indicated mechanism (solid lines; Equations (9)–(12)) to viral titers from ferrets infected with RSV (circles) followed by IAV (squares) after 3 days. Each mechanism included a reduction in the rate of RSV infectivity ($\beta_{\mathrm{RSV}}^{-}$), which was paired with either decreased rates of RSV infected cell clearance ($\delta_{\mathrm{RSV}}^{-}$), IAV infectivity ($\beta_{\mathrm{IAV}}^{-}$), or IAV clearance ($c_{\mathrm{IAV}}^{-}$). Because the RSV dynamics rebounded, these were excluded as possible mechanisms.

Thus, to examine whether increases or decreases in the rates of viral infection, production, clearance, or infected cell clearance could better explain the data, we refit the models together with Equations (13) and/or (14). In total, we evaluated 16 scenarios for a single interaction and, on the basis of those results, up to 18 scenarios for dual interactions where each virus affected the other Table A1 and Table A2). When assuming that the two viruses competed for target cells (Equations (5)–(8)), single interactions that resulted in improved fits (i.e., lower ${\mathrm{AIC}}_{c}$) included an RSV-induced reduction in the rate of IAV infected cell clearance (${\mathrm{ffi}}_{\mathrm{IAV}}^{-}$) or in the rate of IAV clearance ($c_{\mathrm{IAV}}^{-}$) (Table 2; Figure A1A,B). Comparatively, some interactions within the target cell partitioning model (Equations (9)–(12)) led to a rebound of RSV (Figure A2), which were excluded from consideration. The best suggested mechanisms under this hypothesis were either a decrease in the IAV infection rate ($\beta_{\mathrm{IAV}}^{-}$) by RSV together with a decrease in the RSV production rate ($p_{\mathrm{RSV}}^{-}$) by IAV (Table 2, Figure 1C) or an increase in the rate of RSV infected cell clearance by IAV ($\delta_{\mathrm{RSV}}^{+}$; Table A2, Figure A1C).

When allowing for dual interactions, the target cell competition model suggested that there were two sets of mechanisms that provided fits with similar ${\mathrm{AIC}}_{c}$ values as the case when a single interaction was considered (Table 2). Similar to the single interaction results, both sets of mechanisms included a RSV-induced reduction in the rate of IAV infected cell clearance (${\mathrm{ffi}}_{\mathrm{IAV}}^{-}$). This was paired with either an IAV-induced reduction in the rate of RSV production ($p_{\mathrm{RSV}}^{-}$; ${\mathrm{AIC}}_{c}$ value of 175.7 (lowest); Figure 2B) or an IAV-induced increase in the rate of RSV clearance ($c_{\mathrm{RSV}}^{+}$; ${\mathrm{AIC}}_{c}$ value of 175.8; Figure A1B). Allowing for dual interactions in the target cell partitioning model suggested an RSV-mediated reduction in the rate of IAV infectivity (${\mathrm{fi}}_{\mathrm{IAV}}^{-}$) coupled with an IAV-mediated reduction in rate of RSV production ($p_{\mathrm{RSV}}^{-}$; ${\mathrm{AIC}}_{c}$ value of 139.3; Table 2, Figure 2C). The remaining single and double interactions for both models provided fits that were not statistically justifiable (Table A1 and Table A2).

### 3.2. Effect of Infection Timing and Interaction Strength in RSV-IAV Coinfection

Because the order, infection interval, and strength of interaction can influence coinfection dynamics and result in diverse disease phenotypes, we sought to better understand how these metrics alter RSV–IAV coinfection. To achieve this, we evaluated the relative change in total viral burden for each virus (i.e., $\Delta V_{\mathrm{AUC}}$; Equation (15)) across a wide range of infection intervals (0 to 11 days) and interaction strengths (κ; $1\times 10^{-9}$ to $1\times 10^{2}$ (RNA/100 μL)${}^{-1}$) in each model. Here, we focused on the best-fit models with the lowest log-likelihood (Table 2; i.e., $\delta_{\mathrm{IAV}}^{-}$ and $p_{\mathrm{RSV}}^{-}$ in the target cell competition model and $\beta_{\mathrm{IAV}}^{-}$ and $p_{\mathrm{RSV}}^{-}$ in the target cell partitioning model). For the predicted interactions from the earlier analyses, differing the interaction strength (κ) had the strongest effect when the two infections were separated by shorter intervals (i.e., <3 days; Figure 2). When the interval was <3 days in the target cell competition model, a reduction in the RSV burden (up to $\Delta V_{\mathrm{AUC}}$ = −1) and prolonged IAV infection (up to $\Delta V_{\mathrm{AUC}}$ = 15) were observed for a higher interaction strength than the best fit $\delta_{\mathrm{IAV}}^{-}$ value (white stars in Figure 2B). In the target cell partitioning model, the RSV burden was again reduced but the range of interaction strengths was narrower (Figure 2C). Consistent with the data when the IAV infection was initiated 5 or 7 days after RSV [27], the simulations showed that intervals >3 to 4 days after RSV infection resulted in minimal changes in both models (Figure 2B,C). However, only the target cell partitioning led to uninterrupted IAV infection or unchanged IAV viral burden for longer intervals between IAV and RSV infection (Figure 2C).

### 3.3. IAV Coinfection with RV

**Figure 3:**
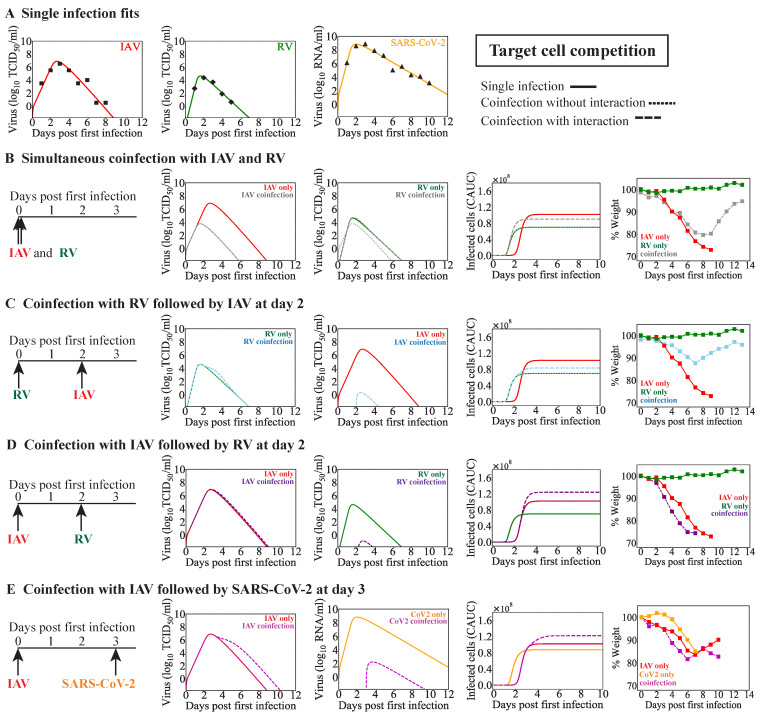
**Model predictions coinfection with IAV and RV or SARS-CoV-2 using the target cell competition model**. (**A**) Fit of the monoinfection model (Equations (1)–(4)) to viral titers from humans infected with IAV (red; solid line) [49], RV (green; solid line) [50], or SARS-CoV-2 (orange; solid line) [51]. (**B**–**E**) Model simulations of the dynamics for monoinfection (solid line) or IAV coinfection with RV or SARS-CoV-2 using the target cell competition model (Equations (5)–(8)) without interaction (dotted line) or with interaction (dashed line). The predicted viral loads and CAUC of the infected cells are shown alongside the weight-loss percentage from infected animals [33,37]. (**B**,**C**) Dynamics of simultaneous coinfection with IAV and RV or RV–IAV with an IAV-mediated decrease in the rate of RV-infected cell clearance ($\delta_{\mathrm{RV}}^{-}$). (**D**) Dynamics of IAV-RV coinfection with an RV-mediated decrease in the rate of IAV infected cell clearance ($\delta_{\mathrm{IAV}}^{-}$). (**E**) Dynamics of IAV-CoV2 coinfection with a CoV2-mediated decrease in the rate of IAV-infected cell clearance ($\delta_{\mathrm{IAV}}^{-}$).

Animals infected with IAV and RV at the same time, or RV two days before IAV, yielded lower weight loss and milder disease severity compared to animals infected with IAV alone (Figure 3). On the other hand, animals infected with IAV 2 days before RV underwent significantly higher weight loss that led to death of all animals by 7 days pi (Figure 3; [33]). To assess the potential mechanisms during IAV coinfection with RV that could lead these empirical observations and provide potential translation to human infection, we first fit the monoinfection model (Equations (1)–(4)) to viral loads from human volunteers infected with IAV [49] or RV [50] (Table 3, Figure 3A). The model fit resulted in different infection kinetic rates for each virus where rate of IAV infection was lower ($\beta_{\mathrm{IAV}}\;=\;2.5\;\times \;10^{-6}$ (TCID${}_{50}$)${}^{-1}$ d${}^{-1}$ vs. $\beta_{\mathrm{RV}}\;=\;1.6\;\times \;10^{-3}$(TCID${}_{50}$)${}^{-1}$ d${}^{-1}$) and the rate of IAV production was higher ($p_{\mathrm{IAV}}\;=\;3.0\;\times \;10^{-1}$/TCID${}_{50}$/cell/day vs. $p_{\mathrm{RV}}\;=\;2.9\;\times \;10^{-3}$/TCID${}_{50}$/cell/day (RV)).

We next used these parameters in the coinfection models (Equations (5)–(8) or Equations (9)–(12)) with or without interaction (Equation (13) or/and Equation (14)) to predict which mechanisms could lead to the distinct disease outcomes observed in the experimental study. Because viral loads were not measured, but weight loss in the infected animals was measured for IAV-RV and RV–IAV coinfections, we compared the estimated cumulative area under the curve (CAUC) of the infected cell dynamics to the weight loss [47], qualitatively matching the magnitude and timing of change. The CAUC of the combined infected cells dynamics of each virus from the coinfection models without any interactions could not recapitulate the weight-loss dynamics for any interval or order, confirming that interactions were occurring.

#### 3.3.1. Simultaneous or Sequential RV–IAV Coinfection

When testing different interaction mechanisms that enhanced or inhibited one virus within the target cell competition model, the model predicted that the mechanism that could lead to the reduced disease severity observed in RV–IAV coinfection (simultaneous or separated by 2 d) was an IAV-mediated decrease in the rate of RV infected cell clearance ($\delta_{\mathrm{RV}}^{-}$; Figure 3B,C). An intermediate signal strength was required for the simultaneous infection ($\kappa_{\delta_{\mathrm{RV}}^{-}}$ = $7\times 10^{-5}$/$\left({\mathrm{TCID}}_{50}/\mathrm{mL}\right)$; Figure 3B) and a larger signal strength was required for a coinfection separated by 2 days ($\kappa_{\delta_{\mathrm{RV}}^{-}}$ = 1/$\left({\mathrm{TCID}}_{50}/\mathrm{mL}\right)$; Figure 3C). In the two scenarios, this could produce similar reductions in the CAUC of the infected cells as the weight-loss patterns in coinfected animals (Figure 3B,C). In addition, when IAV and RV were initiated simultaneously, the model-predicted kinetics showed significant reductions in IAV titers compared to IAV monoinfection (Figure 3B). This was accompanied by a small increase in RV titers around the peak that was due to the IAV-mediated decrease in the rate of RV infected cell clearance. However, in contrast to the simultaneous infection, the model indicated that RV significantly reduced IAV titers when RV was initiated two days before IAV (Figure 3C).

**Figure 4:**
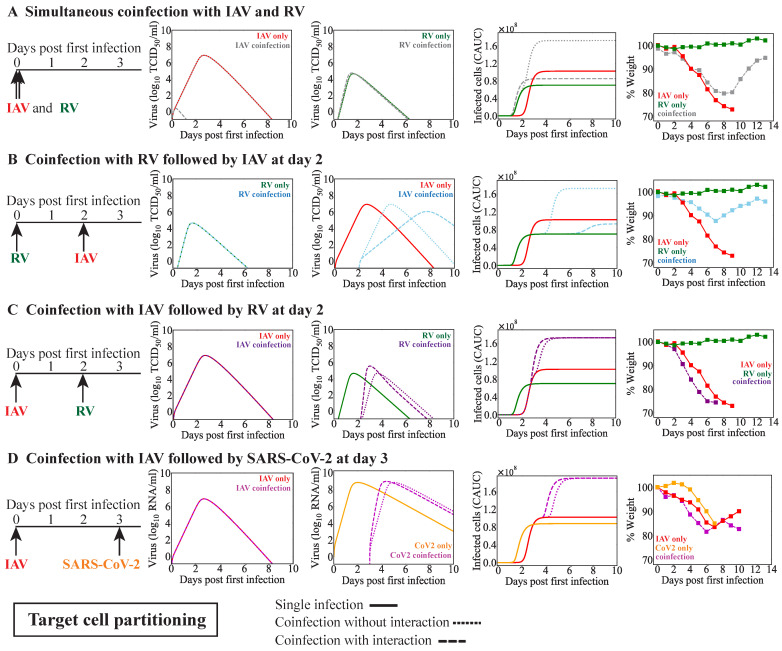
**Model predictions for coinfection with IAV and RV or SARS-CoV-2 using the target cell partitioning model**. Model simulations of the dynamics for monoinfection (solid line) or IAV coinfection with RV or SARS-CoV-2 using the target cell partitioning model (Equations (9)–(12)) without interaction (dotted line) or with interaction (dashed line). The predicted viral loads and CAUC of the infected cells are shown alongside the weight loss percentage from infected animals [33,37]. (**A**) Dynamics of simultaneous coinfection with IAV and RV with an RV-mediated increase in the rate of IAV-infected cell clearance ($\delta_{\mathrm{IAV}}^{+}$) and an IAV-mediated decrease in the rate of RV infected cell clearance ($\delta_{\mathrm{RV}}^{-}$). (**B**) Dynamics of RV-IAV coinfection with reducing the initial number of target cells ($T_{0}$) by 1 $\log_{10}$ for the second infection. (**C**) Dynamics of IAV-RV coinfection with an IAV-mediated increase in the rate of RV production ($p_{\mathrm{RV}}^{+}$). (**D**) Dynamics of IAV-CoV2 coinfection with IAV-mediated increase in the rate of SARS-CoV-2 production ($p_{\mathrm{CoV}2}^{+}$).

**Figure A3:**
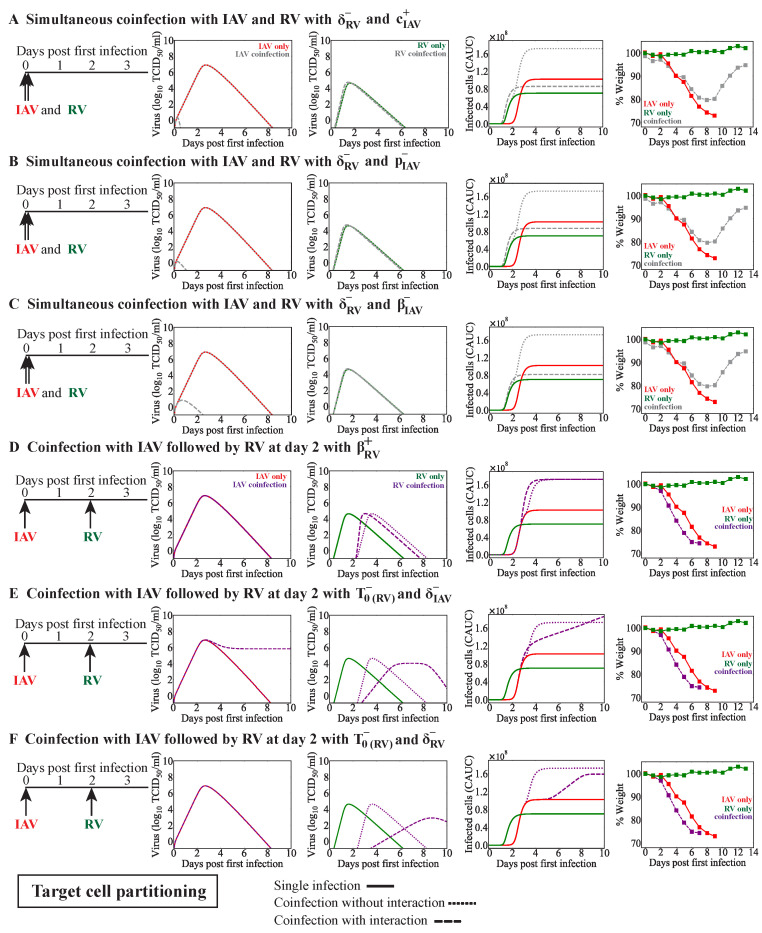
**Model dynamics of alternate mechanisms of IAV-RV coinfection.** Model simulations of the dynamics for monoinfection (solid line) or IAV coinfection with RV using the target cell partitioning model (Equations (9)–(12)) without interaction (dotted line) or with interaction (dashed line). The predicted viral loads and CAUC of the infected cells are shown alongside the percent weight loss from infected animals [33]. Dynamics of simultaneous coinfection with IAV and RV with RV infected cell clearance reduced by IAV ($\delta_{\mathrm{RV}}^{-}$) and (**A**) IAV clearance reduced by RV ($c_{\mathrm{IAV}}^{-}$), (**B**) IAV production reduced by RV ($p_{\mathrm{IAV}}^{-}$), or (**C**) IAV infectivity reduced by RV ($\beta_{\mathrm{IAV}}^{-}$). Dynamics of IAV-RV coinfection with (**D**) RV infectivity increased by IAV ($\beta_{\mathrm{RV}}^{+}$), (**E**) reduced number of target cells ($T_{0}^{-}$) for RV and IAV infected cell clearance reduced by RV ($\delta_{\mathrm{IAV}}^{-}$), or (**F**) reduced number of target cells for RV ($T_{0}^{-}$) and RV infected cell clearance reduced by IAV ($\delta_{\mathrm{RV}}^{-}$).

The reduced rate of RV-infected cell clearance ($\delta_{\mathrm{RV}}^{-}$) was also identified by the target cell partitioning model (Figure 4A,B). However, for a simultaneous infection, this needed to be coupled with an RV-mediated increase in the rate of IAV infected cell clearance ($\delta_{\mathrm{IAV}}^{+}$; $\kappa_{\delta_{\mathrm{IAV}}^{+}}$ = $1\times 10^{2}$/$\left({\mathrm{TCID}}_{50}/\mathrm{mL}\right)$; Figure 4A), an increase in the rate of IAV clearance ($c_{\mathrm{IAV}}^{-}$; $\kappa_{c_{\mathrm{IAV}}^{+}}$ = $1\times 10^{-3}$/$\left({\mathrm{TCID}}_{50}/\mathrm{mL}\right)$;), a decrease in the rate of IAV production ($p_{\mathrm{IAV}}^{-}$; $\kappa_{p_{\mathrm{IAV}}^{-}}$ = $6\times 10^{1}$/$\left({\mathrm{TCID}}_{50}/\mathrm{mL}\right)$; Figure A3B), or a decrease in the rate of IAV infectivity ($\beta_{\mathrm{IAV}}^{-}$; $\kappa_{\beta_{\mathrm{IAV}}^{-}}$ = $6\times 10^{1}$/$\left({\mathrm{TCID}}_{50}/\mathrm{mL}\right)$; Figure A3C) to achieve a consistent CAUC of the infected cells with the reduced weight loss. In all cases, the combined CAUC of the infected cells was reduced to a level below that of an IAV single infection and accompanied with a complete suppression of IAV titers without affecting RV titers.

For RV–IAV coinfection, no single interaction could reproduce the reduced disease severity. Thus, we did not consider dual interactions. However, because viral infections could initiate and/or modify host responses (e.g., Type I interferon, macrophages, and neutrophils) that were not included in our model, and this could translate into a reduced number of susceptible cells that is not automatically created by the target cell partitioning hypothesis, we examined the effect indirectly by reducing the initial number of target cells that were available for the second virus, as in prior studies [47,60]. For RV–IAV coinfection, reducing the initial number of target cells by $1\;\log_{10}$ (i.e., $T_{0}$ = $2\times 10^{7}$ cells for IAV compared to $T_{0}$ = $2\times 10^{8}$ cells for RV; Table 3) was sufficient to reduce the combined CAUC of the infected cells compared to IAV monoinfection (Figure 4B). However, the estimated CAUC of the infected cells deviated from the experimental results at later time points, where the model suggested similar but delayed IAV titers (Figure 4B).

#### 3.3.2. IAV-RV Coinfection

The mechanism that could lead to the increased disease severity observed in IAV-RV coinfection (separated by 2 days) within the target cell competition model was an RV-mediated decrease in the rate of IAV-infected cell clearance ($\delta_{\mathrm{IAV}}^{-}$; Figure 3D). The required signal strength was large ($\kappa_{\delta_{\mathrm{IAV}}^{-}}\;=\;3/\left({\mathrm{TCID}}_{50}/\mathrm{mL}\right)$). Despite the significant model-predicted reduction in RV titers, the small increase in IAV titers was sufficient to create an increase in the combined CAUC of the infected cells that aligned with the increased weight loss observed in coinfected animals (Figure 3D).

In contrast, the target cell partitioning hypothesis alone (i.e., no interactions) led to a higher combined CAUC of the infected cells and alignment with the observed increase in disease severity (Figure 4C). This resulted in similar viral loads as those with the monoinfection for both viruses. However, including an IAV-mediated increase in the rate of RV production ($\kappa_{p_{\mathrm{RV}}^{+}}$ = $1\times 10^{-6}$/$\left({\mathrm{TCID}}_{50}/\mathrm{mL}\right)$; Figure 4C) or the rate of RV infectivity ($\kappa_{\beta_{\mathrm{RV}}^{+}}$ = $1\times 10^{-6}$/(units/mL); Figure A3D) resulted in an earlier increase in the CAUC of infected cells, which matched the timing of the deviation in weight loss slightly better. In the two scenarios, the predicted IAV titer dynamics were similar and unchanged from a monoinfection, and the predicted RV titer dynamics had a similar shape, but were much higher when the production rate was increased (5.51 log10 TCID${}_{50}$/mL vs. 4.63 log10 TCID${}_{50}$/mL). Other possible mechanisms identified by this model included a reduction in the initial number of target cells (i.e., $T_{0}=2\times 10^{7}$ cells for RV compared to $2\times 10^{8}$ cells for IAV; Table 3) coupled with either a reduction in the rate of IAV infected cell clearance by RV ($c_{\mathrm{IAV}}^{-}$; κ = 10/$\left({\mathrm{TCID}}_{50}/\mathrm{mL}\right)$; Figure A3E) or in the rate of RV infected cell clearance by IAV ($\delta_{\mathrm{RV}}^{-}$; κ = 10/$\left({\mathrm{TCID}}_{50}/\mathrm{mL}\right)$; Figure A3F). In the first scenario, the model suggested that IAV titers would remain high for an extended period of time, which created an extended, flat viral-load peak. In the latter case, the model indicated that there would be no changes to IAV titers, and that RV had a slower increase with a lower peak.

### 3.4. IAV Coinfection with SARS-CoV-2

Animals infected with IAV followed 3 days later by SARS-CoV-2 resulted in increased weight loss and more severe disease severity compared to animals infected with IAV or SARS-CoV-2 alone (Figure 3) [37]. To examine the potential interactions between these two viruses, we employed the same approach as above. The results suggested similar mechanisms for enhanced disease severity for the two coinfection models, although the quantitative dynamics was distinct between the models. That is, the target cell competition model predicted a slightly higher combined CAUC of the infected cells when SARS-CoV-2 reduced the rate of IAV infected cell clearance ($\delta_{\mathrm{IAV}}^{-}$) at the signal strength $\kappa_{\delta_{\mathrm{IAV}}^{-}}$ = $2\times 10^{-2}$/(RNA/mL) (Figure 3E) whereas the target cell partitioning without any interaction led to a significantly higher combined CAUC of the infected cells (Figure 4E). Reducing the initial number of target cells (i.e., a $\log_{10}$ reduction [$T_{0}=2\times 10^{7}$ cells]) available to SARS-CoV-2 coupled with a reduction in the rate of SARS-CoV-2-infected cell clearance ($\delta_{\mathrm{CoV}2}^{-}$) by IAV ($\kappa_{\delta_{\mathrm{CoV}2}^{-}}$ = 10/(RNA/mL)) or a reduction in the rate of IAV infected cell clearance ($\delta_{\mathrm{IAV}}^{-}$) by SARS-CoV-2 ($\kappa_{\delta_{\mathrm{IAV}}^{-}}$ = $1\times 10^{-2}$/(RNA/mL)) led to a higher combined CAUC of infected cells. The predicted viral-load dynamics was distinct between the two models. In the target cell competition model, there was a significant reduction in SARS-CoV-2 titers and a delay in the resolution of IAV titers. In contrast, the target cell partitioning model suggested that the SARS-CoV-2 infection was simply delayed.

## 4. Discussion

Respiratory coinfections with multiple viruses are becoming more recognized clinically, particularly in light of the SARS-CoV-2 pandemic. Experimental studies have begun illuminating the outcome heterogeneity, which seems to rely on numerous factors such as the viral pairing, order, and timing of each infection, and specific immune factors. Although viral and immune dynamics during viral–viral coinfections is only beginning to be defined, mathematical models are useful to predict and narrow the spectrum of potential mechanisms, guide new experiments, and help in interpreting clinical, experimental, and epidemiological observations. Our analysis of different viral coinfection scenarios suggests that only a small subset of mechanisms could lead to the alterations in viral loads and/or disease severity observed in animal models (summarized in Table 4).

When a new virus invades within a few days before or after influenza, innate immune responses may be disrupted. The early regulation of Type I IFNs, macrophages, and/or neutrophils, among other innate immune responses, by a virus could impact the dynamics of subsequent infections. Although we did not assess these immune responses directly, we modeled them indirectly in various ways. The target cell competition model automatically assumes that fewer cells are available for infection by the second virus, which could emulate a protective mechanism that reduces the possible infection size for the coinfecting virus. In the target cell partitioning model, we decreased the number of target cells available for the second virus, which was used to mimic lower doses [47,60]. Both approaches assume that some cells are protected or otherwise unavailable for infection, which can be interpreted as the IFN-mediated protection of susceptible cells or an immediate clearance of the virus upon infection due to the activation of macrophages and/or neutrophils by the first virus. We observed the latter phenomenon in an experiment where CD8 T cells were depleted before infection [47]. In that case, innate responses were activated by the CD8 T cell death, which resulted in the partial clearance of the inoculum that emulated a reduced dose, and led to fewer infected cells and thus less virus. This automatically reduced inflammation and weight loss [47], which was similar to the reduced weight loss observed during RV–IAV coinfection [33]. Allowing for fewer susceptible cells within the target cell partitioning model was sufficient to explain the reduced weight loss in RV-IAV coinfected animals, potentially indicating a similar mechanism. While neither our model nor the data were sophisticated enough to specify the exact mechanism, higher IFN-β was detected at 2 days post coinfection, and IAV titers trended slightly lower compared to in the monoinfection [33]. A follow-up study suggested that the protection of IAV-mediated disease by RV was dependent on IFN and that this contributed to the control of neutrophilic inflammation [35]. These data align nicely with our predictions, which may help in connecting the underlying reasoning (i.e., fewer cells becoming infected) with downstream consequences (i.e., reduced immune activation and inflammation).

In addition to potential effects on early host responses, one of the most common mechanisms defined by our analysis was altered rates of infected cell clearance, which may indicate an effect on virus-specific CD8 T-cell responses [47]. In some cases, there was a negative effect, while in others, there was a positive effect (Table 4). Further, there were some scenarios where each virus affected the other virus’ infected cell clearance rate. This may indicate variation in the number, composition, and/or function of epitope-specific T cells, which was observed for other viral pairs (e.g., lymphocytic choriomeningitis (LCMV) and Pichinde (PICV) viruses) [62]. For scenarios where the rates are reduced, it could also indicate that having fewer available target cells from preinfection would have automatically reduced the number of T cells needed to clear the infection. Data defining host responses are important to in helping to distinguish between these possibilities, particularly because CD8 T cells have to be significantly reduced to have a robust impact on viral loads, and their efficacy is dependent on the number of infected cells [47].

**Figure A4:**
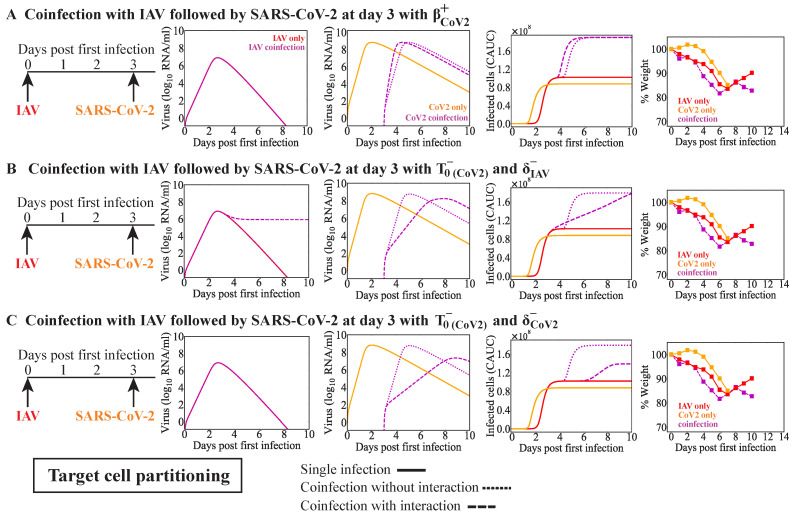
**Model dynamics of alternate mechanisms of IAV-CoV2 coinfection.** Model simulations of the dynamics for monoinfection (solid line) or IAV coinfection with SARS-CoV-2 using the target cell partitioning model (Equations (9)–(12)) without interaction (dotted line) or with interaction (dashed line). The predicted viral loads and CAUC of the infected cells are shown alongside the percent weight loss from infected animals [37]. Dynamics of IAV-CoV2 coinfection with (**A**) SARS-CoV-2 infectivity increased by IAV ($\beta_{\mathrm{CoV}2}^{+}$), (**B**) reduced number of target cells for SARS-CoV-2 ($T_{0}^{-}$) and IAV infected cell clearance reduced by SARS-CoV-2 ($\delta_{\mathrm{IAV}}^{-}$), or (**C**) reduced number of target cells for SARS-CoV-2 ($T_{0}^{-}$) and SARS-CoV-2-infected cell clearance reduced by IAV ($\delta_{\mathrm{CoV}2}^{-}$).

Effects on other infection processes such as the rates of viral infectivity or production were detected for some coinfection scenarios, although this was rarely the sole mechanism (Table 4). Only in IAV-RV and IAV-CoV2 coinfections within the target cell partitioning model were these potential single interactions, and they each resulted in similar viral load dynamics (Figure 4C,D, Figure A3D, and Figure A4A). This is because the type of model used here cannot typically distinguish between the effects of these two processes [63]. There is some evidence that the infectivity of SARS-CoV-2 is increased by IAV but not RSV within cell cultures [41,43]. The underlying mechanism driving this remains unknown, but other studies with IAV and PIV have shown enhanced infection rates where PIV increased cell-to-cell fusion and, thus, spread of IAV [32]. However, another study found that IAV titers were increased while SARS-CoV-2 titers were decreased during IAV-CoV2 coinfection in mice [42]. The discrepancy between these studies, in which one found there was an increase in the number of infected cells, while the other found reduced viral loads, may have been due to the results being obtained in vitro vs. in vivo or to another interaction (e.g., IFN suppression of SARS-CoV-2 or clearance by macrophages). Our analyses suggest that SARS-CoV-2 titers would be reduced within the target cell competition model (Figure A3E,F) or when the number of susceptible cells was reduced within the target cell partitioning model (Figure A4B,C). This may indicate a role for the IAV-activated innate immune response and/or a lower effective dose of SARS-CoV-2.

A limitation of this work is that we used minimal data that were of different types (i.e., viral loads or weight loss) to predict potential mechanisms during viral coinfection. The quantity and type of data used are important because altered viral loads (e.g., as in RSV–IAV coinfection), immune cells, or cytokines do not always directly equate to differences in disease severity. We previously showed this phenomenon where we found that the number of infected cells and inflammation were nonlinearly correlated to disease severity [47]. This was particularly evident in one experiment where CD8 T cells were depleted, which resulted in only small reductions in viral loads, yet large reductions in weight loss. However, as mentioned above, the early immune activation led to a predicted lower effective dose (i.e., fewer cells becoming infected) and, thus, reduced disease severity. Similarly, in RSV–IAV coinfection, IAV titers were slightly increased, yet less weight loss occurred [27]. Thus, the higher viral loads later in infection may be insignificant with respect to severity. This highlights that, while some mechanisms may occur and alter viral loads, they could be distinct from those that yield disease outcomes. Our results from matching the qualitative differences in weight-loss data, which is a measure of disease severity, for IAV coinfection with RV or SARS-CoV-2 may better represent potential mechanisms with measurable differences in outcome. However, many of these also led to predicted differences in viral loads. Some information about mechanism may be able to be deduced from the timing of when weight loss begins to deviate from the monoinfection. In several scenarios, this occurred directly after the initiation of the secondary infection, which suggests that the environment created by the first virus had immediate effects.

The mechanisms suggested by our analysis occasionally differed depending on the underlying model hypothesis (i.e., whether viruses competed for epithelial cells) and, in some cases, resulted in different predicted viral load kinetics. Because most respiratory viruses can infect various types of airway epithelial cells, and replicate in the upper and lower respiratory tracts, it is conceivable that each virus would have ample cells to infect. However, by chance or due to the airway structure, they may enter the same region and interact on a local level. This may lead to cells being coinfected with both viruses, which we did not model explicitly. We indirectly modeled the potential effects of coinfected cells by assuming that the rates of infection and/or production could be different. Interestingly, cellular coinfection was detected during simultaneous infection with RSV and HMPV, where coinfected cells were possible but in small numbers and less likely in the presence of IFN [36]. The same may be true during other coinfections with viruses that are sensitive to IFN antiviral responses. To model the impact of coinfected cells and potential heterogeneity in their prevalence, agent-based models may be better suited than those used here. However, it is important to determine whether and for how long these local effects have a measurable impact during an in vivo infection.

Examining data from viral–viral coinfections using mathematical models allowed for us to reduce the number of possible underlying mechanisms that could result in altered viral load kinetics and/or disease severity. Although the models were relatively simple and lacked investigation into specific host immune responses, the analysis provides the infrastructure to integrate immunological models of higher complexity once data becomes available. Models for some immune responses during respiratory virus infections are already being developed and validated with experimental data (reviewed in [45,64]). Some of the insight from those studies was integrated here and aided our ability to interpret the small amount of experimental data currently available. However, establishing better methods that could predict disease severity (e.g., as in [47]) is critical. Our ability to assess the contribution and timescales of different mechanisms to infection kinetics and outcome should increase as more temporal viral load, immunologic, and pathologic data become available. In addition, the generated hypotheses should aid in experimental design, ultimately leading to a more complete understanding of respiratory virus coinfection.

## Figures and Tables

**Table 1:** **Best-fit parameters for the monoinfection model.** Best-fit parameters obtained from fitting the monoinfection model (Equations (1)–(4)) to viral titers from ferrets intranasally infected with IAV at $3.5\;\log_{10}\;{\mathrm{TCID}}_{50}$ or with RSV at $5.0\;\log_{10}\;\mathrm{PFU}$ [27]. Parameters are reported as the population median and the standard deviation of the associated random effect (ω). The initial numbers of target cells ($T_{0}$) and infected cells ($E_{0}$) were fixed to the indicated values, and the initial number of productively infected cells (I(0)) and the initial viral amount (V(0)) were set to 0.

Parameter	Description	Units	IAV (ω)	RSV (ω)
β	Viral infectivity	(RNA/100 μL)${}^{-1}$ d${}^{-1}$	$3.3\times 10^{-6}$ (0.6)	$1.0\times 10^{-5}$ (0.04)
*k*	Eclipse phase	d${}^{-1}$	5.0	3.0
δ	Infected cell clearance	d${}^{-1}$	0.9 (0.02)	1.1 (1.8)
*p*	Viral production	(RNA/100 μL) cell${}^{-1}$ d${}^{-1}$	$2.5\times 10^{1}$ (0.2)	$3.0\times 10^{-2}$ (0.03)
*c*	Viral clearance	d${}^{-1}$	2.0 (0.06)	1.7 (0.5)
$T_{0}$	Initial target cells	cells	$5\times 10^{7}$	$5\times 10^{7}$
$E_{0}$	Initial eclipse cells	cells	$3.2\times 10^{3}$	$1\times 10^{5}$
$I_{0}$	Initial infected cells	cells	0	0
$V_{0}$	Initial virus	(RNA/100 μL)	0	0

**Table 2:** **Best-fit parameters of the predicted mechanisms of RSV–IAV coinfection.** Best-fit parameters from simulating the coinfection models with no interactions (‘no interaction’) or fitting the target cell competition model (‘competition’; Equations (5)–(8)) or the target cell partitioning model (‘partitioning’; Equations (9)–(12)) with the Equations (13) and/or (14) to viral loads from animals infected with RSV followed by IAV after 3 days. An NLME modeling approach was used and only the strength of interaction (κ) was estimated. Parameters are reported as the population median (κ) with standard deviation of the associated random effect ($\omega_{\kappa }$). Fit quality is reported as log-likelihood (-2LL), ${\mathrm{AIC}}_{c}$, and standard deviation of the residual error (σ). The resulting relative change in total virus ($\Delta V_{\mathrm{AUC}}$) is provided for each scenario.

	Interaction	Effect on IAV		Effect on RSV							
		Strength of	Strength of	Strength of	Strength of	-2LL	${\mathrm{AIC}}_{c}$	$\sigma_{\mathrm{RSV}}$	$\sigma_{\mathrm{IAV}}$	$\Delta V_{\mathrm{AUC}}$	$\Delta V_{\mathrm{AUC}}$
		Enhancement,	Inhibition,	Enhancement,	Inhibition,						
		$\kappa (\omega_{\kappa })$	$\kappa (\omega_{\kappa })$	$\kappa (\omega_{\kappa })$	$\kappa (\omega_{\kappa })$					IAV	RSV
		RNA${}^{-1}$	RNA${}^{-1}$	RNA${}^{-1}$	RNA${}^{-1}$						
**Competition**	No interaction	0	0	0	0	185.0	189.0	0.79	0.81	−0.54	−0.48
	$\delta_{\mathrm{IAV}}^{-}$	–	$7.1\times 10^{-6}$ (0.1)	–	–	166.2	174.2	0.79	0.65	−0.20	−0.48
	$c_{\mathrm{IAV}}^{-}$	–	$6.0\times 10^{-5}$ (0.1)	–	–	167.8	175.8	0.79	0.66	0.74	−0.49
	$\delta_{\mathrm{IAV}}^{-}$ and $p_{\mathrm{RSV}}^{-}$	–	$1.2\times 10^{-5}$ (0.1)	–	$8.7\times 10^{-9}$ (0.2)	163.7	175.7	0.76	0.65	−0.18	−0.66
	$\delta_{\mathrm{IAV}}^{-}$ and $c_{\mathrm{RSV}}^{+}$	–	$1.1\times 10^{-5}$ (0.1)	$4.2\times 10^{-9}$ (0.2)	–	163.8	175.8	0.76	0.65	−0.19	−0.63
**Partitioning**	No interaction	0	0	0	0	181.3	185.3	0.88	0.72	0	0
	$\delta_{\mathrm{RSV}}^{+}$	–	–	$1.9\times 10^{-8}$ (0.04)	–	131.0	139.0	0.40	0.72	0	−0.51
	$\beta_{\mathrm{IAV}}^{-}$ and $p_{\mathrm{RSV}}^{-}$	–	$9.6\times 10^{-5}$ (0.04)	–	$8.8\times 10^{-8}$ (0.1)	127.3	139.3	0.45	0.63	0	−0.53

**Table 3:** **Parameter estimates for human infection with IAV, RV, or SARS-CoV-2.** Parameter estimates from fitting the single virus model in Equations (1)–(4) to viral load data from humans experimentally infected with $4.2\;\log_{10}$${\mathrm{TCID}}_{50}$ IAV [49] or $2.4\;\log_{10}$${\mathrm{TCID}}_{50}$ RV [50], or naturally infected with SARS-CoV-2 [51]. The initial numbers of target cells ($T_{0}$) and infected cells ($E_{0}$) were fixed to the indicated values, and the initial numbers of productively infected cells ($I_{0}$) and the initial virus ($V_{0}$) were set to 0.

Parameter	Description	Units	IAV	RV	SARS-CoV-2
β	Viral infectivity	[V]${}^{-1}$ d${}^{-1}$	$2.5\times 10^{-6}$	$1.6\times 10^{-3}$	$1.4\times 10^{-7}$
*k*	Eclipse phase	d${}^{-1}$	5.0	3.0	3.0
δ	Infected cell clearance	d${}^{-1}$	3.9	5.7	4.5
*p*	Viral production	[V] cell${}^{-1}$ d${}^{-1}$	$3.0\times 10^{-1}$	$2.9\times 10^{-3}$	18.8
*c*	Viral clearance	d${}^{-1}$	3.9	5.6	1.8
$T_{0}$	Initial target cells	cells	$2\times 10^{8}$	$2\times 10^{8}$	$2\times 10^{8}$
$E_{0}$	Initial eclipse cells	cells	100	100	100
$I_{0}$	Initial infected cells	cells	0	0	0
$V_{0}$	Initial virus	[V]	0	0	0
κ	Strength of interaction	[V]${}^{-1}$	See text	See text	See text

[V] indicates ${\mathrm{TCID}}_{50}/\mathrm{mL}$ for IAV and RV, and RNA/mL for SARS-CoV-2.

**Table 4:** **Summary of model-predicted mechanisms resulting in increased or decreased disease severity during viral–viral coinfection.** Summary of model-predicted mechanisms that resulted in altered viral loads (RSV–IAV [27]) or disease severity as quantified by the CAUC of the infected cells [47,48] and measured by animal weight loss (IAV-RV and RV–IAV [33], and IAV-CoV2 [37]).

Model	First Virus	Second Virus	Infection Interval (Days)	Observed Disease Severity (Ref.)	Potential Mechanisms	Figure
**Competition**	RSV	IAV	3	Decreased [27]	IAV-infected cell clearance reduced by RSV and RSV production reduced by IAV	Figure 2B
					IAV infected cell clearance reduced by RSV	Figure A1A
					IAV clearance reduced by RSV	Figure A1A
					IAV infected cell clearance reduced by RSV and RSV clearance increased by IAV	Figure A1B
	IAV	RV	0	Decreased [33]	RV infected cell clearance reduced by IAV	Figure 3B
	RV	IAV	2	Decreased [33]	RV infected cell clearance reduced by IAV	Figure 3C
	IAV	RV	2	Increased [33]	IAV infected cell clearance reduced by RV	Figure 3D
	IAV	SARS-CoV-2	3	Increased [37]	IAV infected cell clearance reduced by SARS-CoV-2	Figure 3E
**Partitioning**	RSV	IAV	3	Decreased [27]	IAV infectivity reduced by RSV and RSV production reduced by IAV	Figure 2C
					RSV-infected cell clearance increased by IAV	Figure A1C
	IAV	RV	0	Decreased [33]	RV infected cell clearance reduced by IAV and IAV infected cell clearance increased by RV	Figure 4A
					RV infected cell clearance reduced by IAV and IAV clearance reduced by RV	Figure A3A
					RV infected cell clearance reduced by IAV and IAV production reduced by RV	Figure A3B
					RV infected cell clearance reduced by IAV and IAV infectivity reduced by RV	Figure A3C
	RV	IAV	2	Decreased [33]	Reduced number of target cells for IAV	Figure 4B
	IAV	RV	2	Increased [33]	RV production increased by IAV	Figure 4C
					No interaction	Figure 4C
					RV infectivity increased by IAV	Figure A3D
					Reduced number of target cells for RV and IAV infected cell clearance reduced by RV	Figure A3E
					Reduced number of target cells for RV and RV infected cell clearance reduced by IAV	Figure A3F
	IAV	SARS-CoV-2	3	Increased [37]	SARS-CoV-2 production increased by IAV	Figure 4D
					No interaction	Figure 4D
					SARS-CoV-2 infectivity increased by IAV	Figure A4A
					Reduced number of target cells for SARS-CoV-2 and IAV infected cell clearance reduced by SARS-CoV-2	Figure A4B
					Reduced number of target cells for SARS-CoV-2 and SARS-CoV-2-infected cell clearance reduced by IAV	Figure A4C

## Data Availability

No new data were created or analyzed in this study. Data sharing is not applicable to this article.

## Appendix A

### Appendix A.1. Complete Results from Fitting the Coinfection Models to Data from RSV-IAV Coinfection in Ferrets

The complete set of best-fit parameters obtained from fitting the coinfection models (‘competition’, Equations (5)–(8); ‘partitioning’, Equations (9)–(12)) with the Equations (13) and/or (14) to viral loads from animals infected with RSV followed by IAV after 3 days is in Table A1 and Table A2. The top models are highlighted in gray and the dynamics are in Figure 2 and Figure A1. Models with a predicted RSV rebound (Figure A2) were excluded.

**Table A1:** **Full parameter results from fitting the target cell competition model to RSV-IAV coinfection in ferrets.** Parameters from simulating (‘no interaction’) or fitting the target cell competition model (‘competition’; Equations (5)–(8)) with the Equations (13) and/or (14) to viral loads from animals infected with RSV followed by IAV after 3 days. An NLME modeling approach was used and only the strength of interaction (κ) was estimated. Parameters are reported as the population median (κ) with standard deviation of the associated random effect ($\omega_{\kappa }$). Fit quality is reported as log-likelihood (-2LL), ${\mathrm{AIC}}_{c}$, and standard deviation of the residual error (σ). The best models and predicted mechanisms are highlighted in gray and summarized in Table 1.

Interaction	IAV		RSV		-2LL	${\mathrm{AIC}}_{c}$	$\sigma_{\mathrm{RSV}}$	$\sigma_{\mathrm{IAV}}$
	Strength of	Strength of	Strength of	Strength of				
	Enhancement,	Inhibition,	Enhancement,	Inhibition,				
	$\kappa (\omega_{\kappa })$	$\kappa (\omega_{\kappa })$	$\kappa (\omega_{\kappa })$	$\kappa (\omega_{\kappa })$				
	**RNA** ${}^{-1}$	**RNA** ${}^{-1}$	**RNA** ${}^{-1}$	**RNA** ${}^{-1}$				
No interaction	0	0	0	0	185.0	189.0	0.79	0.81
Single interaction								
$\beta_{\mathrm{IAV}}^{+}$	$1.9\times 10^{-18}$ (6.5)	–	–	–	185.0	193.0	0.79	0.81
$p_{\mathrm{IAV}}^{+}$	$3.4\times 10^{-12}$ (2.5)	–	–	–	185.0	193.0	0.79	0.81
$\delta_{\mathrm{IAV}}^{+}$	$6.7\times 10^{-11}$ (8.5)	–	–	–	185.0	193.0	0.79	0.81
$c_{\mathrm{IAV}}^{+}$	$2.2\times 10^{-10}$ (3.5)	–	–	–	185.0	193.0	0.79	0.81
$\beta_{\mathrm{IAV}}^{-}$	–	$4.3\times 10^{-18}$ (6.9)	–	–	185.0	193.0	0.79	0.81
$p_{\mathrm{IAV}}^{-}$	–	$8.5\times 10^{-17}$ (5.3)	–	–	185.0	193.0	0.79	0.81
$\delta_{\mathrm{IAV}}^{-}$	–	$7.1\times 10^{-6}$ (0.1)	–	–	166.2	174.2	0.79	0.65
$c_{\mathrm{IAV}}^{-}$	–	$6.0\times 10^{-5}$ (0.1)	–	–	167.8	175.8	0.79	0.66
$\beta_{\mathrm{RSV}}^{+}$	–	–	$1.6\times 10^{-16}$ (4.5)	–	185.0	193.0	0.79	0.81
$p_{\mathrm{RSV}}^{+}$	–	–	$1.2\times 10^{-13}$ (2.0)	–	185.0	193.0	0.79	0.81
$\delta_{\mathrm{RSV}}^{+}$	–	–	$3.3\times 10^{-14}$ (2.0)	–	185.0	193.0	0.79	0.81
$c_{\mathrm{RSV}}^{+}$	–	–	$8.6\times 10^{-9}$ (0.2)	–	182.5	190.5	0.76	0.81
$\beta_{\mathrm{RSV}}^{-}$	–	–	–	$1.4\times 10^{-5}$ (2.3)	182.0	190.0	0.81	0.76
$p_{\mathrm{RSV}}^{-}$	–	–	–	$1.6\times 10^{-8}$ (0.4)	182.5	190.5	0.76	0.80
$\delta_{\mathrm{RSV}}^{-}$	–	–	–	$1.1\times 10^{-9}$ (0.9)	183.7	191.7	0.77	0.81
$c_{\mathrm{RSV}}^{-}$	–	–	–	$2.2\times 10^{-13}$ (2.2)	185.0	193.0	0.79	0.81
Double interactions								
$\delta_{\mathrm{IAV}}^{-}$ and $\beta_{\mathrm{RSV}}^{-}$	–	$6.5\times 10^{-6}$ (0.1)	–	$7.5\times 10^{-17}$ (11.1)	166.1	178.1	0.79	0.65
$\delta_{\mathrm{IAV}}^{-}$ and$p_{\mathrm{RSV}}^{-}$	–	$1.2\times 10^{-5}$ (0.1)	–	$8.7\times 10^{-9}$ (0.2)	163.7	175.7	0.76	0.65
$\delta_{\mathrm{IAV}}^{-}$ and $\delta_{\mathrm{RSV}}^{+}$	–	$6.6\times 10^{-6}$ (0.1)	$3.6\times 10^{-8}$ (5.3)	–	166.1	178.1	0.79	0.65
$\delta_{\mathrm{IAV}}^{-}$ and $c_{\mathrm{RSV}}^{+}$	–	$1.1\times 10^{-5}$ (0.1)	$4.2\times 10^{-9}$ (0.2)	–	163.8	175.8	0.76	0.65
$c_{\mathrm{IAV}}^{-}$ and $\beta_{\mathrm{RSV}}^{-}$	–	$5.6\times 10^{-5}$ (0.1)	–	$8.6\times 10^{-21}$ (16.6)	167.7	179.7	0.79	0.66
$c_{\mathrm{IAV}}^{-}$and $p_{\mathrm{RSV}}^{-}$	–	$1.0\times 10^{-4}$ (0.1)	–	$3.8\times 10^{-9}$ (0.3)	165.5	177.5	0.76	0.66
$c_{\mathrm{IAV}}^{-}$ and $\delta_{\mathrm{RSV}}^{+}$	–	$6.0\times 10^{-5}$ (0.1)	$1.4\times 10^{-16}$ (5.1)	–	167.7	179.7	0.79	0.66
$c_{\mathrm{IAV}}^{-}$ and $c_{\mathrm{RSV}}^{+}$	–	$9.5\times 10^{-5}$ (0.1)	$1.9\times 10^{-9}$ (0.3)	–	165.7	177.7	0.77	0.66

**Table A2:** **Full parameter results from fitting the target cell partitioning model to RSV-IAV coinfection in ferrets.** Parameters from simulating (‘no interaction’) or fitting the target cell partitioning model (‘competition’; Equations (5)–(8)) with the Equations (13) and/or (14) to viral loads from animals infected with RSV followed by IAV after 3 days. An NLME modeling approach was used and only the strength of interaction (κ) was estimated. Parameters are reported as the population median (κ) with standard deviation of the associated random effect ($\omega_{\kappa }$). Fit quality is reported as log-likelihood (-2LL), ${\mathrm{AIC}}_{c}$, and standard deviation of the residual error (σ). The best models and predicted mechanisms are highlighted in gray and summarized in Table 1. Although some models resulted in the lower AIC${}_{c}$ values, they were excluded because they produced a viral load rebound.

Interaction	IAV		RSV		-2LL	${\mathrm{AIC}}_{c}$	$\sigma_{\mathrm{RSV}}$	$\sigma_{\mathrm{IAV}}$
	Strength of	Strength of	Strength of	Strength of				
	Enhancement,	Inhibition,	Enhancement,	Inhibition,				
	$\kappa (\omega_{\kappa })$	$\kappa (\omega_{\kappa })$	$\kappa (\omega_{\kappa })$	$\kappa (\omega_{\kappa })$				
	**RNA** ${}^{-1}$	**RNA** ${}^{-1}$	**RNA** ${}^{-1}$	**RNA** ${}^{-1}$				
No interaction	0	0	0	0	181.3	185.3	0.88	0.72
Single interaction								
$\beta_{\mathrm{IAV}}^{+}$	$6.0\times 10^{-12}$ (2.9)	–	–	–	181.3	189.3	0.88	0.72
$p_{\mathrm{IAV}}^{+}$	$3.5\times 10^{-11}$ (2.2)	–	–	–	181.3	189.3	0.88	0.72
$\delta_{\mathrm{IAV}}^{+}$	$2.3\times 10^{-15}$ (2.3)	–	–	–	181.3	189.3	0.88	0.72
$c_{\mathrm{IAV}}^{+}$	$7.1\times 10^{-16}$ (2.2)	–	–	–	181.3	189.3	0.88	0.72
$\beta_{\mathrm{IAV}}^{-}$	–	$1.3\times 10^{-6}$ (0.90)	–	–	180.5	188.5	0.88	0.71
$p_{\mathrm{IAV}}^{-}$	–	$6.3\times 10^{-5}$ (0.06)	–	–	168.6	176.6	0.88	0.62
$\delta_{\mathrm{IAV}}^{-}$	–	$7.0\times 10^{-7}$ (0.2)	–	–	178.3	186.3	0.88	0.70
$c_{\mathrm{IAV}}^{-}$	–	$4.9\times 10^{-7}$ (1.9)	–	–	180.7	188.7	0.88	0.71
$\beta_{\mathrm{RSV}}^{+}$	–	–	$7.4\times 10^{-7}$ (1.0)	–	178.8	186.8	0.84	0.72
$p_{\mathrm{RSV}}^{+}$	–	–	$1.4\times 10^{-34}$ (12.6)	–	181.2	189.2	0.88	0.72
$\delta_{\mathrm{RSV}}^{+}$	–	–	$1.9\times 10^{-8}$ (0.04)	–	131.0	139.0	0.40	0.72
$c_{\mathrm{RSV}}^{+}$	–	–	$7.1\times 10^{-9}$ (0.10)	–	169.2	177.2	0.72	0.72
$\beta_{\mathrm{RSV}}^{-}$	–	–	–	$1.8\times 10^{-5}$ (0.1)	128.6	136.6	0.38	0.72
$p_{\mathrm{RSV}}^{-}$	–	–	–	$1.0\times 10^{-8}$ (0.1)	169.7	177.7	0.73	0.72
$\delta_{\mathrm{RSV}}^{-}$	–	–	–	$6.8\times 10^{-19}$ (4.1)	181.3	189.3	0.88	0.72
$c_{\mathrm{RSV}}^{-}$	–	–	–	$8.8\times 10^{-18}$ (6.5)	181.3	189.3	0.88	0.72
Double interactions								
$\delta_{\mathrm{IAV}}^{-}$ and $\beta_{\mathrm{RSV}}^{-}$	–	$2.1\times 10^{-6}$ (0.3)	–	$1.2\times 10^{-5}$ (0.1)	125.9	137.9	0.38	0.70
$\delta_{\mathrm{IAV}}^{-}$ and $p_{\mathrm{RSV}}^{-}$	–	$2.5\times 10^{-6}$ (0.2)	–	$1.0\times 10^{-8}$ (0.08)	164.9	176.9	0.70	0.70
$\delta_{\mathrm{IAV}}^{-}$ and $\delta_{\mathrm{RSV}}^{+}$	–	$2.8\times 10^{-31}$ (10.9)	$1.9\times 10^{-8}$ (0.05)	–	131.0	143.0	0.40	0.72
$\delta_{\mathrm{IAV}}^{-}$ and $c_{\mathrm{RSV}}^{+}$	–	$2.4\times 10^{-6}$ (0.1)	$6.3\times 10^{-9}$ (0.1)	–	164.0	176.0	0.70	0.70
$c_{\mathrm{IAV}}^{-}$ and $\beta_{\mathrm{RSV}}^{-}$	–	$2.8\times 10^{-16}$ (7.2)	–	$1.9\times 10^{-5}$ (0.2)	128.5	140.5	0.38	0.72
$c_{\mathrm{IAV}}^{-}$ and $p_{\mathrm{RSV}}^{-}$	–	$2.3\times 10^{-5}$ (0.3)	–	$6.1\times 10^{-9}$ (0.1)	163.9	175.9	0.66	0.72
$c_{\mathrm{IAV}}^{-}$ and $\delta_{\mathrm{RSV}}^{+}$	–	$6.0\times 10^{-17}$ (3.0)	$1.9\times 10^{-8}$ (0.03)	–	130.9	142.9	0.40	0.72
$c_{\mathrm{IAV}}^{-}$ and $c_{\mathrm{RSV}}^{+}$	–	$2.6\times 10^{-5}$ (0.2)	$3.6\times 10^{-9}$ (0.07)	–	163.1	175.1	0.65	0.73
$\beta_{\mathrm{IAV}}^{-}$ and $\beta_{\mathrm{RSV}}^{-}$	–	$5.5\times 10^{-5}$ (0.4)	–	$1.2\times 10^{-5}$ (0.1)	120.2	132.2	0.40	0.62
$\beta_{\mathrm{IAV}}^{-}$ and $p_{\mathrm{RSV}}^{-}$	–	$9.6\times 10^{-5}$ (0.04)	–	$8.8\times 10^{-8}$ (0.1)	127.3	139.3	0.45	0.63

### Appendix A.2. Alternate Interactions from Simulating the Target Cell Partitioning Model for IAV Coinfection with RV or SARS-CoV-2

Dynamics of the target cell partitioning model (Equations (9)–(12)) with alternate mechanisms for simultaneous IAV and RV coinfection (Figure A3A–C), IAV-RV coinfection (Figure A3D–F), and IAV-CoV2 coinfection (Figure A4A–C).

## References

[1] Nickbakhsh S., Mair C., Matthews L., Reeve R., Johnson P.C., Thorburn F., Von Wissmann B., Reynolds A., McMenamin J., Gunson R.N. (2019). Virus–virus interactions impact the population dynamics of influenza and the common cold. Proc. Natl. Acad. Sci. USA.

[2] Nowak M.D., Sordillo E.M., Gitman M.R., Mondolfi A.E.P. (2020). Coinfection in SARS-CoV-2 infected patients: Where are influenza virus and rhinovirus/enterovirus?. J. Med. Virol..

[3] Sanz I., Perez D., Rojo S., Domínguez-Gil M., de Lejarazu R.O., Eiros J.M. (2022). Coinfections of influenza and other respiratory viruses are associated to children. An. Pediatr..

[4] Park J.S., Chu S.Y., Shin Y.Y., Ryu I.K., Tang C.L., Choi J., Kim H.B., Kim C.K. (2019). Comparison of clinical severity between single-and coinfections of respiratory syncytial virus and influenza virus with common respiratory viruses. Allergy Asthma Respir. Dis..

[5] Nolan V.G., Arnold S.R., Bramley A.M., Ampofo K., Williams D.J., Grijalva C.G., Self W.H., Anderson E.J., Wunderink R.G., Edwards K.M. (2018). Etiology and impact of coinfections in children hospitalized with community-acquired pneumonia. J. Infect. Dis..

[6] Pacheco G.A., Gálvez N., Soto J.A., Andrade C.A., Kalergis A.M. (2021). Bacterial and Viral Coinfections with the Human Respiratory Syncytial Virus. Microorganisms.

[7] Calcagno A., Ghisetti V., Burdino E., Trunfio M., Allice T., Boglione L., Bonora S., Di Perri G. (2021). Co-infection with other respiratory pathogens in COVID-19 patients. Clin. Microbiol. Infect..

[8] Meskill S.D., O’Bryant S.C. (2020). Respiratory virus co-infection in acute respiratory infections in children. Curr. Infect. Dis. Rep..

[9] Cheng Y., Ma J., Wang H., Wang X., Hu Z., Li H., Zhang H., Liu X. (2021). Co-infection of influenza A virus and SARS-CoV-2: A retrospective cohort study. J. Med. Virol..

[10] Rotzén-Östlund M., Eriksson M., Tiveljung Lindell A., Allander T., Zweygberg Wirgart B., Grillner L. (2014). Children with multiple viral respiratory infections are older than those with single viruses. Acta Paediatr..

[11] Fayyadh T.K., Ma F., Qin C., Zhang X., Li W., Zhang X.E., Zhang Z., Cui Z. (2017). Simultaneous detection of multiple viruses in their coinfected cells using multicolour imaging with self-assembled quantum dot probes. Microchim. Acta.

[12] Choi S.H., Chung J.W., Kim H.R. (2015). Clinical relevance of multiple respiratory virus detection in adult patients with acute respiratory illness. J. Clin. Microbiol..

[13] Jain S., Williams D.J., Arnold S.R., Ampofo K., Bramley A.M., Reed C., Stockmann C., Anderson E.J., Grijalva C.G., Self W.H. (2015). Community-acquired pneumonia requiring hospitalization among US children. N. Engl. J. Med..

[14] Jain S., Self W.H., Wunderink R.G., Fakhran S., Balk R., Bramley A.M., Reed C., Grijalva C.G., Anderson E.J., Courtney D.M. (2015). Community-acquired pneumonia requiring hospitalization among US adults. N. Engl. J. Med..

[15] Goka E.A., Vallely P.J., Mutton K.J., Klapper P.E. (2014). Single and multiple respiratory virus infections and severity of respiratory disease: A systematic review. Paediatr. Respir. Rev..

[16] Martin E.T., Kuypers J., Wald A., Englund J.A. (2012). Multiple vs. single virus respiratory infections: Viral load and clinical disease severity in hospitalized children. Influenza Other Respir. Viruses.

[17] Aberle J.H., Aberle S.W., Pracher E., Hutter H.P., Kundi M., Popow-Kraupp T. (2005). Single vs. dual respiratory virus infections in hospitalized infants: Impact on clinical course of disease and interferon-*γ* response. Pediatr. Infect. Dis. J..

[18] Stefanska I., Romanowska M., Donevski S., Gawryluk D., Brydak L.B. (2013). Co-infections with influenza and other respiratory viruses. Respiratory Regulation—The Molecular Approach.

[19] Scotta M.C., Chakr V.C.B.G., de Moura A., Becker R.G., de Souza A.P.D., Jones M.H., Pinto L.A., Sarria E.E., Pitrez P.M., Stein R.T. (2016). Respiratory viral coinfection and disease severity in children: A systematic review and meta-analysis. J. Clin. Virol..

[20] Greer R.M., McErlean P., Arden K.E., Faux C.E., Nitsche A., Lambert S.B., Nissen M.D., Sloots T.P., Mackay I.M. (2009). Do rhinoviruses reduce the probability of viral co-detection during acute respiratory tract infections?. J. Clin. Virol..

[21] Cebey-López M., Herberg J., Pardo-Seco J., Gómez-Carballa A., Martinón-Torres N., Salas A., Martinón-Sánchez J.M., Gormley S., Sumner E., Fink C. (2015). Viral co-infections in pediatric patients hospitalized with lower tract acute respiratory infections. PLoS ONE.

[22] Zhong P., Zhang H., Chen X., Lv F. (2019). Clinical characteristics of the lower respiratory tract infection caused by a single infection or coinfection of the human parainfluenza virus in children. J. Med. Virol..

[23] Goka E., Vallely P., Mutton K., Klapper P. (2013). Influenza A viruses dual and multiple infections with other respiratory viruses and risk of hospitalization and mortality. Influenza Other Respir. Viruses.

[24] Zhang G., Hu Y., Wang H., Zhang L., Bao Y., Zhou X. (2012). High incidence of multiple viral infections identified in upper respiratory tract infected children under three years of age in Shanghai, China. PLoS ONE.

[25] Brand H.K., de Groot R., Galama J.M., Brouwer M.L., Teuwen K., Hermans P.W., Melchers W.J., Warris A. (2012). Infection with multiple viruses is not associated with increased disease severity in children with bronchiolitis. Pediatr. Pulmonol..

[26] Shinjoh M., Omoe K., Saito N., Matsuo N., Nerome K. (2000). In vitro growth profiles of respiratory syncytial virus in the presence of influenza virus. Acta Virol..

[27] Chan K.F., Carolan L.A., Korenkov D., Druce J., McCaw J., Reading P.C., Barr I.G., Laurie K.L. (2018). Investigating viral interference between influenza A virus and human respiratory syncytial virus in a ferret model of infection. J. Infect. Dis..

[28] Essaidi-Laziosi M., Geiser J., Huang S., Constant S., Kaiser L., Tapparel C. (2020). Interferon-dependent and respiratory virus-specific interference in dual infections of airway epithelia. Sci. Rep..

[29] Drori Y., Jacob-Hirsch J., Pando R., Glatman-Freedman A., Friedman N., Mendelson E., Mandelboim M. (2020). Influenza A virus inhibits RSV infection via a two-wave expression of IFIT proteins. Viruses.

[30] Hartwig S.M., Miller A.M., Varga S.M. (2022). Respiratory Syncytial Virus Provides Protection against a Subsequent Influenza A Virus Infection. J. Immunol..

[31] Haney J., Vijayakrishnan S., Streetley J., Dee K., Goldfarb D.M., Clarke M., Mullin M., Carter S.D., Bhella D., Murcia P.R. (2022). Coinfection by influenza A virus and respiratory syncytial virus produces hybrid virus particles. Nat. Microbiol..

[32] Goto H., Ihira H., Morishita K., Tsuchiya M., Ohta K., Yumine N., Tsurudome M., Nishio M. (2016). Enhanced growth of influenza A virus by coinfection with human parainfluenza virus type 2. Med. Microbiol. Immunol..

[33] Gonzalez A.J., Ijezie E.C., Balemba O.B., Miura T.A. (2018). Attenuation of influenza A virus disease severity by viral coinfection in a mouse model. J. Virol..

[34] Wu A., Mihaylova V.T., Landry M.L., Foxman E.F. (2020). Interference between rhinovirus and influenza A virus: A clinical data analysis and experimental infection study. Lancet Microbe.

[35] Van Leuven J.T., Gonzalez A.J., Ijezie E.C., Wixom A.Q., Clary J.L., Naranjo M.N., Ridenhour B.J., Miller C.R., Miura T.A. (2021). Rhinovirus reduces the severity of subsequent respiratory viral infections by interferon-dependent and-independent mechanisms. Msphere.

[36] Geiser J., Boivin G., Huang S., Constant S., Kaiser L., Tapparel C., Essaidi-Laziosi M. (2021). RSV and HMPV Infections in 3D Tissue Cultures: Mechanisms Involved in Virus-Host and Virus-Virus Interactions. Viruses.

[37] Clark J.J., Penrice-Randal R., Sharma P., Kipar A., Dong X., Davidson A.D., Williamson M.K., Matthews D.A., Turtle L., Prince T. (2020). Sequential infection with influenza A virus followed by severe acute respiratory syndrome coronavirus 2 (SARS-CoV-2) leads to more severe disease and encephalitis in a mouse model of COVID-19. bioRxiv.

[38] Huang Y., Skarlupka A.L., Jang H., Blas-Machado U., Holladay N., Hogan R.J., Ross T.M. (2022). SARS-CoV-2 and Influenza A virus Co-infections in Ferrets. J. Virol..

[39] Zhang A.J., Lee A.C.Y., Chan J.F.W., Liu F., Li C., Chen Y., Chu H., Lau S.Y., Wang P., Chan C.C.S. (2021). Coinfection by Severe Acute Respiratory Syndrome Coronavirus 2 and Influenza A (H1N1) pdm09 Virus Enhances the Severity of Pneumonia in Golden Syrian Hamsters. Clin. Infect. Dis..

[40] Bao L., Deng W., Qi F., Lv Q., Song Z., Liu J., Gao H., Wei Q., Yu P., Xu Y. (2021). Sequential infection with H1N1 and SARS-CoV-2 aggravated COVID-19 pathogenesis in a mammalian model, and co-vaccination as an effective method of prevention of COVID-19 and influenza. Signal Transduct. Target. Ther..

[41] Bai L., Zhao Y., Dong J., Liang S., Guo M., Liu X., Wang X., Huang Z., Sun X., Zhang Z. (2021). Coinfection with influenza A virus enhances SARS-CoV-2 infectivity. Cell Res..

[42] Achdout H., Vitner E.B., Politi B., Melamed S., Yahalom-Ronen Y., Tamir H., Erez N., Avraham R., Weiss S., Cherry L. (2021). Increased lethality in influenza and SARS-CoV-2 coinfection is prevented by influenza immunity but not SARS-CoV-2 immunity. Nat. Commun..

[43] Fage C., Hénaut M., Carbonneau J., Piret J., Boivin G. (2022). Influenza A (H1N1) pdm09 Virus but Not Respiratory Syncytial Virus Interferes with SARS-CoV-2 Replication during Sequential Infections in Human Nasal Epithelial Cells. Viruses.

[44] Dee K., Goldfarb D.M., Haney J., Amat J.A., Herder V., Stewart M., Szemiel A.M., Baguelin M., Murcia P.R. (2021). Human rhinovirus infection blocks severe acute respiratory syndrome coronavirus 2 replication within the respiratory epithelium: Implications for COVID-19 epidemiology. J. Infect. Dis..

[45] Smith A.M. (2018). Host-pathogen kinetics during influenza infection and coinfection: Insights from predictive modeling. Immunol. Rev..

[46] Pinky L., Dobrovolny H.M. (2016). Coinfections of the respiratory tract: Viral competition for resources. PLoS ONE.

[47] Myers M.A., Smith A.P., Lane L.C., Moquin D.J., Vogel P., Woolard S., Smith A.M. (2021). Dynamically linking influenza virus infection with lung injury to predict disease severity. eLife.

[48] Smith A.P., Lane L.C., Ramirez Zuniga I., Moquin D.M., Vogel P., Smith A.M. (2022). Increased virus dissemination leads to enhanced lung injury but not inflammation during influenza-associated secondary bacterial infection. FEMS Microbes.

[49] Murphy B.R., Rennels M.B., Douglas Jr R.G., Betts R.F., Couch R.B., Cate Jr T.R., Chanock R.M., Kendal A.P., Maassab H.F., Suwanagool S. (1980). Evaluation of influenza A/Hong Kong/123/77 (H1N1) ts-1A2 and cold-adapted recombinant viruses in seronegative adult volunteers. Infect. Immun..

[50] Hendley J.O., Gwaltney Jr J.M. (2004). Viral titers in nasal lining fluid compared to viral titers in nasal washes during experimental rhinovirus infection. J. Clin. Virol..

[51] Wölfel R., Corman V.M., Guggemos W., Seilmaier M., Zange S., Müller M.A., Niemeyer D., Jones T.C., Vollmar P., Rothe C. (2020). Virological assessment of hospitalized patients with COVID-2019. Nature.

[52] Smith A.M., Perelson A.S. (2011). Influenza A virus infection kinetics: Quantitative data and models. Wiley Interdiscip. Rev. Syst. Biol. Med..

[53] Matrosovich M., Herrler G., Klenk H.D. (2015). Sialic acid receptors of viruses. Top. Curr. Chem..

[54] Johansen M., Irving A., Montagutelli X., Tate M., Rudloff I., Nold M., Hansbro N., Kim R., Donovan C., Liu G. (2020). Animal and translational models of SARS-CoV-2 infection and COVID-19. Mucosal Immunol..

[55] Kogure T., Suzuki T., Takahashi T., Miyamoto D., Hidari K.I., Guo C.T., Ito T., Kawaoka Y., Suzuki Y. (2006). Human trachea primary epithelial cells express both sialyl (*α*2-3) Gal receptor for human parainfluenza virus type 1 and avian influenza viruses, and sialyl (*α*2-6) Gal receptor for human influenza viruses. Glycoconj. J..

[56] Bohmwald K., Galvez N., Canedo-Marroquín G., Pizarro-Ortega M.S., Andrade-Parra C., Gómez-Santander F., Kalergis A.M. (2019). Contribution of cytokines to tissue damage during human respiratory syncytial virus infection. Front. Immunol..

[57] Shinya K., Ebina M., Yamada S., Ono M., Kasai N., Kawaoka Y. (2006). Influenza virus receptors in the human airway. Nature.

[58] Antony F. (2020). Monolix Version 2019R1. http://lixoft.com/products/monolix/.

[59] Smith A.P., Moquin D.J., Bernhauerova V., Smith A.M. (2018). Influenza virus infection model with density dependence supports biphasic viral decay. Front. Microbiol..

[60] Pinky L., Burke C.W., Russell C.J., Smith A.M. (2021). Quantifying dose-, strain-, and tissue-specific kinetics of parainfluenza virus infection. PLoS Comput. Biol..

[61] Akaike H. (1998). Information theory and an extension of the maximum likelihood principle. Selected Papers of Hirotugu Akaike.

[62] Kenney L.L., Cornberg M., Chen A.T., Emonet S., de la Torre J.C., Selin L.K. (2015). Increased immune response variability during simultaneous viral coinfection leads to unpredictability in CD8 T cell immunity and pathogenesis. J. Virol..

[63] Smith A.M., Adler F.R., Perelson A.S. (2010). An accurate two-phase approximate solution to an acute viral infection model. J. Math. Biol..

[64] Smith A.M. (2018). Validated models of immune response to virus infection. Curr. Opin. Syst. Biol..

